# Wheel Running Improves Motor Function and Spinal Cord Plasticity in Mice With Genetic Absence of the Corticospinal Tract

**DOI:** 10.3389/fncel.2019.00106

**Published:** 2019-03-19

**Authors:** Wei Zhang, Bin Yang, Huandi Weng, Tao Liu, Lingling Shi, Panpan Yu, Kwok-Fai So, Yibo Qu, Libing Zhou

**Affiliations:** ^1^Guangdong-Hongkong-Macau Institute of CNS Regeneration, Ministry of Education CNS Regeneration Collaborative Joint Laboratory, Jinan University, Guangzhou, China; ^2^Co-innovation Center of Neuroregeneration, Nantong University, Jiangsu, China; ^3^Key Laboratory of Neuroscience, School of Basic Medical Sciences, Institute of Neuroscience, The Second Affiliated Hospital, Guangzhou Medical University, Guangzhou, China

**Keywords:** exercise, corticospinal tract, animal model, oligodendrogenesis, neural plasticity, transcriptomics

## Abstract

Our previous studies showed that mutant mice with congenital absence of the corticospinal tract (CST) undergo spontaneous remodeling of motor networks to partially compensate for absent CST function. Here, we asked whether voluntary wheel running could further improve locomotor plasticity in CST-deficient mice. Adult mutant mice were randomly allocated to a “runners” group with free access to a wheel, or a “non-runners” group with no access to a wheel. In comparison with non-runners, there was a significant motor improvement including fine movement, grip strength, decreased footslip errors in runners after 8-week training, which was supported by the elevated amplitude of electromyography recording and increased neuromuscular junctions in the biceps. In runners, terminal ramifications of monoaminergic and rubrospinal descending axons were significantly increased in spinal segments after 12 weeks of exercise compared to non-runners. 5-ethynyl-2′-deoxyuridine (EDU) labeling showed that proliferating cells, 90% of which were Olig2-positive oligodendrocyte progenitors, were 4.8-fold more abundant in runners than in non-runners. In 8-week runners, RNAseq analysis of spinal samples identified 404 genes up-regulated and 398 genes down-regulated, and 69 differently expressed genes involved in signal transduction, among which the NF-κB, PI3K-Akt and cyclic AMP (cAMP) signaling were three top pathways. Twelve-week training induced a significant elevation of postsynaptic density protein 95 (PSD95), synaptophysin 38 and myelin basic protein (MBP), but not of brain derived neurotrophic factor (BDNF), glial cell line-derived neurotrophic factor (GDNF) and insulin like growth factor-1 (IGF-1). Thus, locomotor training activates multiple signaling pathways, contributes to neural plasticity and functional improvement, and might palliate locomotor deficits in patients.

## Introduction

The corticospinal tract (CST) is a main descending axonal bundle for motor control. Its axons originate from corticospinal motor neurons in cortical layer V and innervate different segments of the spinal cord. In the human, damage to the CST is a leading cause of motor disability in spinal cord injury (SCI), amyotrophic lateral sclerosis, cerebral palsy and related diseases. Although wiring by intact axons and re-innervation by axonal sprouting contributes to functional recovery, the limited growth ability of the adult central nervous system remains a formidable impediment to regeneration and treatment (Jin et al., 2015; Carmichael et al., 2017).

Motor training is used to improve motor skills in patients with SCI or cerebral palsy (Willerslev-Olsen et al., 2014, 2015; Wu et al., 2017; Kuczynski et al., 2018), and may assist in restoring locomotor ability by facilitating neural plasticity, attenuating inflammation and/or improving tolerance to physical activity (Cobianchi et al., 2017). There is some debate as to what animal models best mimics cerebral palsy and allow treatment evaluation (Clowry et al., 2014). Interestingly, an intrinsic plasticity of locomotor networks (Schulz, 2006) is known to persist through life in rodents (Kleim et al., 2003) and probably in human (Vahdat et al., 2015). Several studies confirm the benefit of specific locomotor training and/or stimulation of activity-dependent plasticity (Martin et al., 2007, 2011; Dunlop, 2008; Lynskey et al., 2008; Ilha et al., 2011). Others suggest that exercise induces an up-regulation of neurotrophins (NTs) such as brain derived neurotrophic factor (BDNF), nerve growth factor (NGF), NT-3, and NT-4, and thereby may facilitate neural plasticity and functional recovery, possibly through cyclic AMP (cAMP) signaling (Hannila and Filbin, 2008; Jung et al., 2014; Lee and Soya, 2017; Tian et al., 2018).

Using *Celsr3* conditional gene inactivation, we generated a mouse model in which the CST is congenitally absent (Zhou et al., 2008). Although wiring of alternative motor networks provides significant functional compensation, deficits in fine motor behavior and muscle tone are clearly present (Han et al., 2015). We wondered whether voluntary exercise training could increase further compensatory mechanisms in those mice. To test this, animals were trained in a running wheel for 8–12 weeks, after which we evaluated locomotor performances and studied changes in the spinal cord microenvironment, the morphological maturity of motor-related components, and the plastic changes of spinal networks. Our data provide rational arguments for therapy by exercise in patients with disorders of spinal cord or CST, such as in cerebral palsy.

## Materials and Methods

### Animals

Animal procedures were approved by the Laboratory Animal Ethics Committee at Jinan University (ref file: 20150302007). *Emx1–Cre;Celsr3^+/–^* males were crossed with *Celsr3*^f/f^ females to obtain *Emx1–Cre;Celsr3*^f/-^ mice, thereafter referred as “mutant” (Zhou et al., 2008). Adult mutant mice (6–8 weeks old, 22–25 g) were individually housed and randomly allocated to runners and non-runners groups. Littermates with genotypes *Emx1–Cre;Celsr3*^f/+^ or *Celsr3*^f/-^ were used as controls. Females and males were used indiscriminately and all animals were kept under a 12-h light-dark cycle (light on at 7:00 a.m. and off at 7:00 p.m.) with *ad libitum* access to food and water. Mice were given free access to a silent spinner running wheel (diameter: 10.8 cm, width: 4.7 cm, rod spacing: 0.7 cm, rod diameter: 0.3 cm), which was locked in non runners. ER-4000 energizer/receivers (Mini Mitter, Bend, OR, USA) were placed above the cage to monitor motor activity, and the data were acquired using Vital View. Data were analyzed using Exceland Actiview to generate periodograms and actograms (Harkin et al., 2002; Eckel-Mahan and Sassone-Corsi, 2015).

### Behavioral Tests

Mice underwent test sessions at approximately the same time each day, and eight animals were used in each group.

#### Grid Tests

The grid area (32 × 15 cm) has 11/11 mm openings. Behavior was recorded using a camera placed under the grid, in order to assess stepping errors (i.e., “footfaults”). Foot slips during the first 100 steps were scored when the paw missed a rung and the animal lost balance, or when the paw slipped off during weight bearing. For each limb, footfaults were counted and compared to the overall step number made by that limb.

#### Grip Strength Tests

Grip tests were performed every 4 weeks with a 47,200 Grip Strength Meter (Ugo Basile, Italy). This method consists of measuring the grip force using a dynamometer while the mouse is being pulled by the tail (Alamri et al., 2018). Mice were tested five times in succession, with a 3-min rest, and three best results were averaged.

#### Food-Pellet Taking Tests

Skilled motor function was assessed by testing food pellet handling. After fasting for 24 h, animals were videotaped to record food handling. Irvine, Beatties and Bresnahan (IBB) scores ranging from 0 to 9 were used to estimate forelimb usage, based on joint position, object support, digit movement and grasping technique (Irvine et al., 2010).

#### Home-Cage Tests

Walking distance was assessed by home-cage tests (Jhuang et al., 2010). At the end of the 12-week exercise period, runner and non-runner mice were placed for 5 days in individual cages (27 × 17 × 16 cm) inside cabinets with constant temperature and humidity, equipped with infrared cameras to monitor behavioral activity. The first 3 days served as an acclimatization period, and during the last 2 days, behavior was recorded and analyzed using the HomeCageScan 3.0 software.

### Immunostaining

At the end of the 12-week experimental period, mice were deeply anesthetized with 4% tribromoethanol and perfused intracardially with 4% paraformaldehyde in 0.01 M phosphate-buffered saline (PBS, pH 7.4). Spinal C5–C7 segments and biceps (six animals in each group) were collected and post-fixed in the same fixative overnight, and then immersed in 10%-20%-30% sucrose until they sank. Ten micrometer transverse spinal sections and 40-μm horizontal biceps sections were prepared with a sliding microtome (Leica). Sections were kept in 12-well plates at 4°C. After washing with PBS, sections were blocked in 10% goat serum plus 3% bovine serum albumin for 2 h, and incubated with the primary antibodies overnight at 4°C. The primary antibodies included: goat anti-choline acetyltransferase (anti-ChAT; 1:500; AB144p, Millipore), rabbit anti-oligodendrocyte transcription factor 2 (anti-Olig2; 1:2,000, AB9610, Millipore), mouse anti-serotonin (anti-5-HT; 1:500, ab6336, Abcam) and rabbit anti-tyrosine hydroxylase (anti-TH; 1:500; AB152, Millipore). Sections were rinsed in 0.01 M PBS and incubated with the secondary fluorescent antibodies (Alexa Fluor 488 or 546; 1:1,000, A21202/A21206/A11055, Thermo Fisher) for 2 h. α-bungarotoxin conjugated to Alexa Fluor 546 (α-BT; 1:1,000, T1175, Molecular Probes) was used to label acetylcholine receptor clusters.

### Electrophysiology

To determine whether running wheel exercise modifies the properties of muscle, evoked electromyography was measured. At the end of the 12-week experimental period, an investigator blind to groups submitted mice to light sedation using propofol and inserted concentric needle electrodes in the biceps. A square pulse (50–100 μA stimulus of 100 μs at 0.13 Hz) was applied to musculocutaneous nerves and recordings were measured using Keypoint Portable (Dantec Biomed, Denmark). The stimulation was gradually increased from 50 μA to 100 μA until the maximal amplitude appeared just before large motor responses were evoked (typically between 80 μA and 100 μA). The maximum amplitude was measured and the latency was calculated from the initiation of pulse stimulus to the beginning of the response peak. The mean amplitude and wave width were calculated for each animal based on average tracing of more than 10 sweeps. Six animals were used in each group.

### 5-Ethynyl-2′-Deoxyuridine (EDU) Labeling *in vivo*

5-ethynyl-2′-deoxyuridine (EDU; Sigma, St. Louis, MI, USA) was dissolved at 10 mg/ml in 0.9% NaCl, and sterilized by filtration at 0.2 μm. Mice (*n* = 4 in each group) received one intraperitoneal injection of 50 mg/kg body weight per day for three consecutive days during the last of the 12 training weeks. Twenty-four hours after the last injection, mice were perfused with 4% paraformaldehyde and EDU was detected using the Alexa Fluor 555 Click-iT detection kit (Invitrogen). One of six series of adjacent C5–C7 spinal sections were used to count EDU-labeled cells.

### Biotinylated Dextran Amine (BDA) Tracing

Animals (*n* = 3 in each group) were anesthetized and placed in a head holder (68004, RWD Life Science Co. Ltd., China). For anterograde tracing, 1 μl of biotinylated dextran amine (BDA; 10,000 MW, 10% in PBS, pH 7.4, Molecular Probes) was injected in the right red nucleus (3.49 mm posterior to the Bregma, 0.89 mm lateral to the midline, 3.94 mm ventral to the skull surface). Fourteen days later, animals were anesthetized and perfused with 4% paraformaldehyde. Spinal cords were postfixed overnight at 4°C. The distribution of BDA labeling was detected in 40 μm thick transverse sections of C5–C7 spinal segments using a BDA-10000 Neuronal Tracer kit (N-7167, molecular probes) and the density of axons was evaluated as described (Jin et al., 2015). Briefly, we drew a horizontal line through the central canal and across the lateral rim of the gray matter. Vertical lines were then drawn to divide the horizontal line into 100 μm intervals, starting from the central canal to the lateral rim. Crosses between axons and the vertical lines were counted in each section.

### Bio-Plex Pro™ Cytokine Assays

After 12-week running, blood was collected from the retro-orbital sinus. Thereafter, the mice (*n* = 3 in each group) were killed by decapitation, and C5–C7 spinal segments and biceps were quickly collected. Lysates from biceps and spinal cords were prepared using RIPA solution containing Phenylmethanesulfonyl fluoride (PMSF; 1:100; P8340-1; Solarbio Bioscience and Technology), Protease Inhibitor Cocktail (1:100, 539137-10vlcn, Millipore) and Phosphatase Inhibitor Cocktail (1:100, 539131, Calbiochem). Bio-Plex Pro™ cytokine assays (BIO-RAD, Hercules, CA, USA) were used to measure cytokines in blood, spinal cords and muscles by using a 23-plex test kit, which included IL-1α, IL-1β, IL-2, IL-3, IL-4, IL-5, IL-6, IL-9, IL-10, IL-12 (p40), IL-12 (p70), IL-13, IL-17, Eotaxin, G-CSF, GM-CSF, IFN-γ, KC, MCP-1, MIP-1α, MIP-1β, RANTES and TNF-α.

### Western Blotting

After 12-week exercise, animals (*n* = 3 in each group) were killed and proteins were extracted from C5 to C7 spinal segments, which were separated by 10% sodium dodecyl sulfate polyacrylamide gel electrophoresis. Proteins were then transferred to nitrocellulose and the blots were probed with anti-myelin basic protein (anti-MBP) rabbit polyclonal antibody (1:1,000, ab40390, Abcam), anti-TH rabbit polyclonal antibody (1:1,000, AB152, Millipore), anti-synaptophysin mouse polyclonal antibody (SY38; 1:500, ab8049, Abcam), anti-postsynaptic density-95 (anti-PSD-95) rabbit polyclonal antibody (1:500, 516900, Invitrogen), anti-insulin like growth factor-1 (anti-IGF-1) rabbit polyclonal antibody (1:2,000, ab9572, Abcam), anti-glial cell line-derived neurotrophic factor (anti-GDNF) rabbit polyclonal antibody (1:500, ab18956, Abcam), anti-BDNF (1:500, ab108319, Abcam), anti-β actin rabbit polyclonal antibody (1:5,000; ab8227, Abcam), anti-β tubulin rabbit polyclonal antibody (1:5,000; ab6046, Abcam). Peroxidase anti-rabbit IgG (1:5,000, ab6721, Abcam) and peroxidase anti-mouse IgG (1:10,000, Vector Laboratories) were used as secondary antibodies. Immunoreactivity was detected using an enhanced chemiluminescence (ECL) detection kit (1705061, Bio-Rad).

### RNAseq Analysis

Eight-week runner and non-runner mutant mice (three animals from each group) were sacrificed and C5–C7 spinal segments were quickly dissected under a dissecting microscope. Total RNA from every six mice was extracted using a TRIzol Plus RNA Purification Kit (Cat. No. 12183018A, Invitrogen Life Technologies). The quality and quantity of the purified RNA were assessed using Agilent 2100 Bioanalyzer. RNA samples with high purity (28S/18S > 2.0) and high integrity (RIN > 8.0) were used for cDNA library construction. Eight microgram RNA from each sample was used for RNAseq (Illumina HiSeq™ 2500, BGI) at the Schengen platform^1^. The method based on fragments per kilobase of exon per million fragments mapped (FPKM) was used to calculate gene expression. FPKM values were averaged for the three runners and three non-runners, and Poisson distribution analysis was performed to identify differentially expressed genes (DEGs) in the two groups. A FPKM filtering cutoff was taken as less than 0.5 in at least one of the six samples to remove genes with low abundance. Comparing the runner and the non-runner groups, transcripts were selected for further analysis by taking the cutoff of a fold change (FC) higher than 1.15 or less than 0.85 and the cutoff of *P*-value less than 0.05. The KEGG database was used to identify enriched pathways in DEGs relatively to the whole genome background (Kanehisa et al., 2008).

### Statistical Analysis

Results are presented as mean ± SEM. Comparisons of different time points or groups were performed using the Student–Newman Keuls (*q* test) or Bonferroni correction. Single comparison between runner and non-runner animals at each time point was done using two-independent samples Student’s *t*-test. Statistical significance was expressed as * or **P < 0.05 or *P* < 0.01, respectively.

## Results

### Mutant Mice Improve Motor Performances After Training

**Figure 1:**
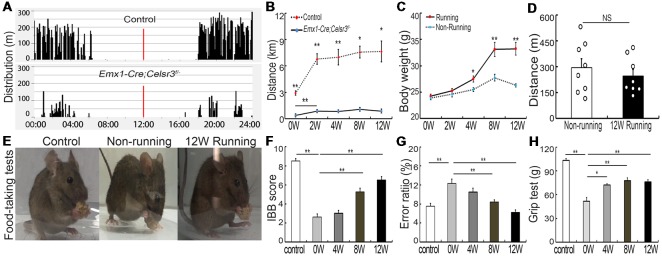
Wheel running improves motor performance in *Emx1-Cre;Celsr3^f/-^* mutants. **(A)** Nycthemeral activity chart in control and mutant mice shows that mutants run during the dark period like controls, albeit less extensively. **(B)** Distance covered during 24 h by running in the wheel is lower in mutants than controls. *P < 0.05; **P < 0.01; *n* = 8. **(C)** Body weight increases in runners vs. non-runners after 8 weeks (8W) of exercise. *P < 0.05; **P < 0.01; *n* = 8. **(D)** Distance covered by walking in the cage is comparable in non-runners and runners after 12 weeks (12W) of running. Each circle represents the data from one animal. *P* > 0.05; *n* = 8; NS, not significant. **(E)** Examples of food-taking test performances in control, non-runner and runner mutants. Most 12-week runner mutants (6/8) could easily lift food pellets from the floor, like control animals (8/8), whereas non-runner mutants (0/8) failed to do so. **(F)** Irvine, Beatties and Bresnahan (IBB) scores in runners display a significant increase after 8 weeks (8W) of exercise and peak at 12 weeks (12W) compared to non-runners. **P < 0.01; *n* = 8. **(G)** Gait coordination, estimated by grid tests, shows higher error rate in the mutant than in the control, with gradual improvement during exercise. **P < 0.01; *n* = 8. **(H)** Muscle strength assessed by grasping tests. Forelimb strength in mutants is lower than in control mice and gradually increases with training. *P < 0.05; **P < 0.01; *n* = 8.

In contrast to free walking, voluntary wheel running implies a series of complex movements and motor-skill learning (Willuhn and Steiner, 2008). Control and mutant mice displayed comparable daily rhythms, with more activity in the dark than in the light (Figure 1A). After 2 weeks of running wheel exercise, the 24-h movement distance was significantly increased compared to that of the day of the experiment start in control (6,787.42 ± 601.47 m vs. 2,913.82 ± 238.07 m; *p* = 0.001, *t* = 5.988, *df* = 14) and mutant mice (855.75 ± 54.85 vs. 376.75 ± 66.17 m; *p* = 0.001, *t* = 5.573, *df* = 14), suggesting that both mice have the ability to learn this kind of complex movements. However, the total 24-h running distance in the wheel was significantly shorter in mutants than in control mice (Figure 1B), indicating a deficit of complex movements in mutants. In contrast to non-runner mutants, voluntary wheel running contributed to an increase of body weight, showing a significant difference in runner mutants after 4-week exercise (Figure 1C; *p* = 0.011, *t* = 2.935, *df* = 14). However, the 24-h moving distance was comparable between non-runner and runner mutants (295.33 ± 52.59 m vs. 247.28 ± 36.91 m; *p* = 0.467, *t* = 0.748, *df* = 14) at the end of 12-week exercise assessed by home-cage tests (Figure 1D; *n* = 8 in each group), indicating running exercise cannot rescue hyperactivity in mutant animals as reported (Han et al., 2015).

*Emx1-Cre;Celsr3^f/-^* mutant mice have impaired skilled-motor function of forepaws (Han et al., 2015). To assess whether running exercise improves skilled movements, animals were submitted to food-pellet taking tests. At the end of 12-week exercise, most mutants (6/8) could easily grasp and lift small pellets away from the floor like control animals (8/8), whereas mutant non-runners (0/8) could not (Figure 1E). This was further confirmed by IBB scores: a significant increase appeared after 8 weeks running and peaked after 12 weeks running (6.50 ± 0.34, *n* = 8) compared to non-runner mutants (2.63 ± 0.32, *n* = 8), but still did not reach the scores of control animals (8.50 ± 0.27, *n* = 8; Figure 1F). In addition, wheel running improved gait coordination, as indicated by the significant decrease of the error ratios (falling steps to total steps) after 8 weeks running (Figure 1G; *p* = 0.007, *t* = 3.146, *df* = 14), a 31.4% decrease compared to mutants before running. In control animals, there were no significant changes in food-pellet taking tests or grid tests after 12-week wheel running (data not shown), thus we focused our study in mutant animals thereafter.

Compared to control mice, the forelimb grasping strength was significantly lower in mutant mice, and it increased gradually after wheel running (Figure 1H, *n* = 8), with the increase of 39.68% (*p* = 0.001, *t* = 4.34, *df* = 14) at 4 weeks and 50.06% (*p* = 0.001, *t* = 4.615, *df* = 14) at 8 weeks compared to that before running exercise.

**Figure 2:**
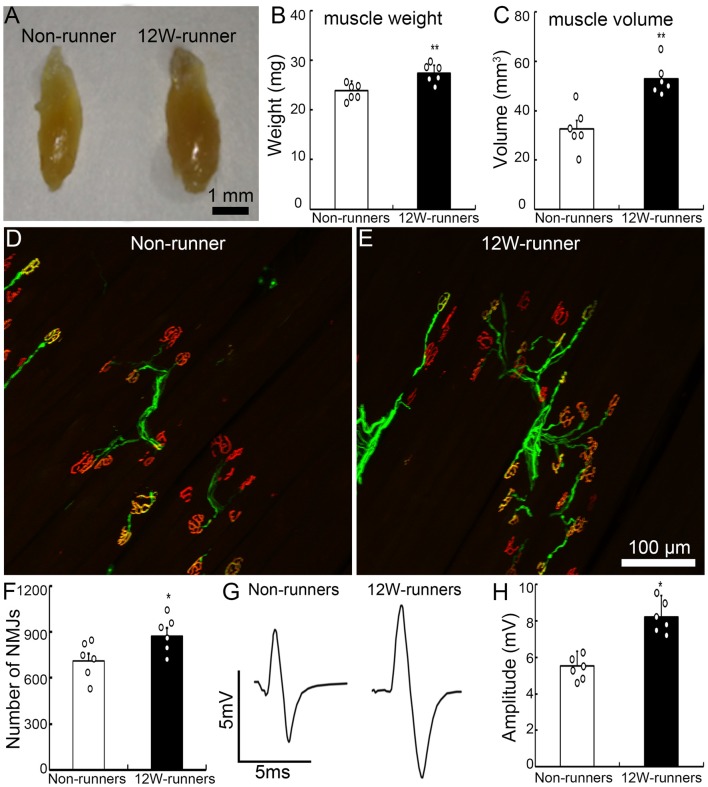
Exercise fosters reorganization of neuromuscular junctions. **(A–C)** Twelve-week (12W) exercise increases the wet weight and volume of biceps in mutant mice. Each circle represents the data from one animal. **P < 0.01; *n* = 6. **(D–F)** Examples of neuromuscular junctions in a non-runner **(D)** and a runner after 12-week exercise **(E)**, illustrating the higher number of nerve terminals (neurofilaments, green) and acetylcholine receptor clusters (α-BT, red) in runners, quantified in **(F)**. Each circle represents the data from one animal. *P < 0.05; *n* = 6. **(G,H)** Electromyographic comparison. The peak-to-peak electromyography amplitude is increased in runners (**G**, right) vs. non-runners (**G**, left), as quantified in **(H)**. Each circle represents the data from one animal. *P < 0.05; *n* = 6.

Forelimb function depends on elbow movements driven by the biceps brachii. After 12 weeks of exercise, the weight and volume of biceps brachii were significantly increased compared to mutant non-runners (Figures 2A–C; 23.93 ± 0.76 mg vs. 27.45 ± 0.63 mg, *p* = 0.005, *t* = 3.549, *df* = 10; 32.66 ± 3.51 mm^3^ vs. 53.00 ± 2.65 mm^3^, *p* = 0.001, *t* = 4.619). To test whether exercise triggers reorganization of spinal motor axons in muscles, we studied neuromuscular junctions by double staining with anti-neurofilament antibodies for axon terminals and α-BT for acetylcholine receptor clusters. After 12 weeks of exercise, the number of motor axonal termini surrounded by α-BT-positive acetylcholine receptor clusters was significantly increased in the runners compared to the non-runners group (*p* = 0.04, *t* = 2.358, *df* = 10; Figures 2D,E), and there was an increase of 22.8% of neuromuscular junctions compared to those in the non-runners group (Figure 2F). We recorded electromyography of biceps by stimulating the musculocutaneous nerve and found that the peak-to-peak electromyography amplitude was significantly increased in the runners after 12 weeks of exercise compared to the non-runners (Figures 2G,H; *p* = 0.001, *t* = 6.374, *df* = 10). Taken together, the data indicate that voluntary running fosters the reorganization of neuromuscular junctions and improves the electrophysiological function and skilled-motor performances.

### Training Results in Increased Ramifications of Rubrospinal and Monoaminergic Descending Axons

**Figure 3:**
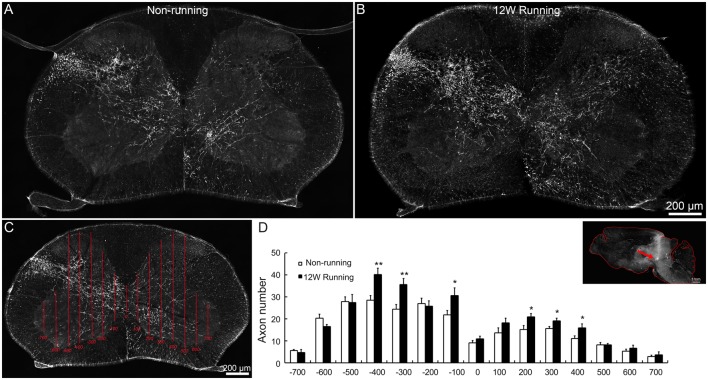
Projections of red nuclei to the spinal cord are increased after training. **(A,B)** Upon biotinylated dextran amine (BDA) injections in the red nucleus, well stained axons are widely distributed in the dorsolateral white matter and the entire gray matter contralateral to the injection side, and some cross the midline and innervate the ipsilateral gray matter, both in runners **(A)** and non-runners **(B)**. **(C,D)** Quantitative assessment of axon extension shows that, after 12 weeks (12W) of training, the number of grid crossing is significantly increased at contralateral (indicated as −400, −300 and −100 μm away from the midline) and ipsilateral levels (indicated as 200, 300 and 400 μm away from the midline) in runners in comparison to non-runners. *P < 0.05; **P < 0.01; *n* = 3 for each group. The insert in **(D)** showed the injection site (arrow).

To assess whether voluntary wheel running induces compensatory reorganization of rubrospinal axons in mutant spinal cords, we injected the anterograde tracer BDA in red nuclei and analyzed the density of labeled fibers in spinal C5–C7 segments. In transverse sections, well stained axons were widely distributed in the dorsolateral white matter and the gray matter contralateral to the injection side; some of them crossed the midline and innervated the ipsilateral gray matter, in samples from the runners and non-runners (Figures 3A,B). We quantified the fiber density as reported (Jin et al., 2015; Figure 3C) and found that, in runners after 12 weeks of exercise, crossing events were significantly increased contralaterally (indicated as −400, −300 and −100 μm away from the midline) and ipsilaterally (indicated as 200, 300 and 400 μm away from the midline) compared to non-runners (Figure 3D; *n* = 3 animals in each group).

**Figure 4:**
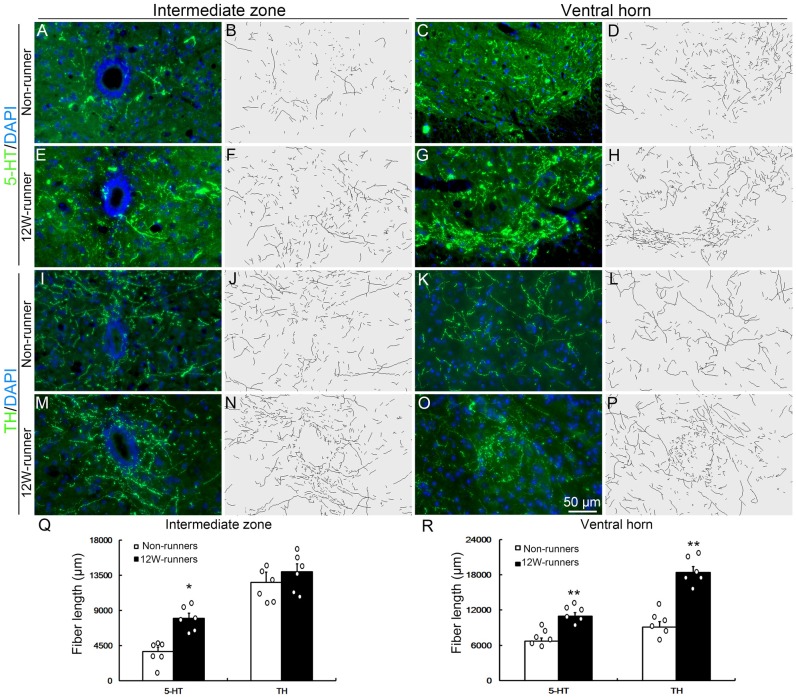
Monoaminergic fibers in the spinal cord are increased after training. **(A–P)** Intermediate zone and ventral horn of spinal cord at C5–C7 segments, stained with anti-serotonin (anti-5-TH, **A–H**) and anti-tyrosine hydroxylase (anti-TH, **I–P**) antibodies, with outline of fiber patterns upon 3D reconstruction. **(Q,R)** Quantification of fiber length in intermediate zone and ventral horn. Each circle represents the data from one animal. *P < 0.05; **P < 0.01; *n* = 6 for each group.

Besides corticospinal and rubrospinal projections, spinal segments receive serotonergic inputs from the pons and dopaminergic inputs from the midbrain (Jordan et al., 2008). The monoaminergic information is implicated in the initiation of locomotion under normal conditions, and the reorganization of their axons in the spinal cord contributes to functional recovery after SCI (Schmidt and Jordan, 2000; Carelli et al., 2017). To investigate whether voluntary wheel running influences monoaminergic innervation, C5−C7 transverse spinal sections were immunostained with anti-5-HT and anti-TH antibodies, followed with tri-dimensional reconstruction of fiber distribution. 5-HT- and TH-positive fibers were identified in the intermediate zone and the ventral horn in both groups (Figures 4A–P). In the intermediate zone, there was a significant increase of serotoninergic (*p* = 0.01, *t* = 4.882, *df* = 10), but not of dopaminergic fibers (*p* = 0.230, *t* = 1.278, *df* = 10) after 12 weeks of exercise in runners compared to non-runners (Figures 4B,F,J,N,Q). In the ventral horn, the density of serotoninergic and dopaminergic fibers was dramatically increased after 12-week running exercise (Figures 4D,H,L,P,R; *p* = 0.001, *t* = 4.882 and *p* = 0.001, *t* = 6.857, respectively).

**Figure 5:**
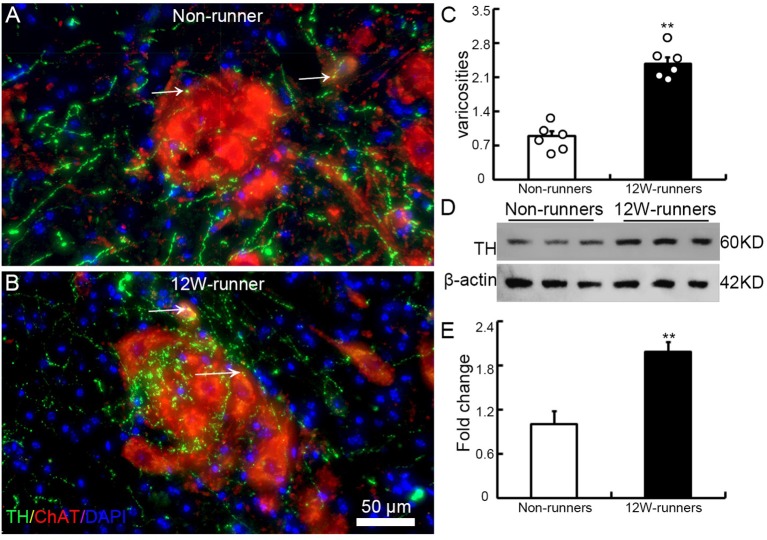
Training induces increased contacts between dopaminergic axons and spinal motor neurons. **(A,B)** Illustration of contacts between TH-positive varicosities and cholinergic neurons in a non-runner **(A)** and a runner after training **(B)** the white arrows indicate “contacting varicosities between 5-HT fibers and ChAT-positive neurons”. **(C)** Quantification of TH-positive varicosities. Each circle represents the data from one animal. **P < 0.01; *n* = 6 for each group. **(D)** Western blot analysis of TH protein content in three non-runners and three runners, relatively to β-actin control, with quantification. **(E)** Statistics of protein levels. **P < 0.01.

To examine whether the increased monoaminergic projections make more contacts with spinal motor neurons, we performed double immunostaining with anti-ChAT antibodies to label spinal motor neurons, and anti-TH and anti-5-HT antibodies in C5–C7 transverse spinal sections. We did not observe more contacts between 5-HT-positive fibers and ChAT-positive cells (data not shown). By contrast, TH-positive fibers came into close proximity to spinal motor neurons, particularly in samples from runners (Figures 5A,B). We quantified the number of TH-positive varicosities adjacent to cholinergic motor neurons or dendrites. The criteria were taken by visualizing an obvious varicose structure (more than 2-fold of axon diameter) along the axons and no visible space between the varicosity and the neuron when viewed at high magnification by confocal microscopy (Takeoka et al., 2009, 2010). In 12-week runner mutant mice, there was a significant increase of varicosities between TH-positive fibers and cholinergic spinal motor neurons (Figure 5C; *n* = 6 mice in each group). In agreement with this, western blot analysis showed that the TH protein level in runners’ spinal cords was about 2-fold higher than in non-runners (Figures 5D,E; *p* = 0.001, *t* = 7.84, *df* = 4).

### Training Activates Oligodendrogenesis in the Spinal Cord

**Figure 6:**
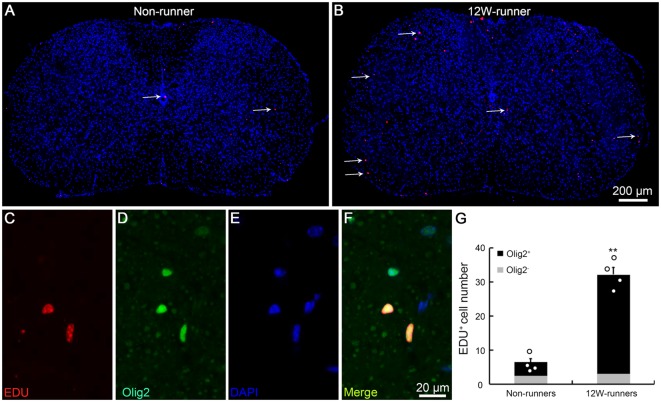
Exercise activates neurogenesis in the spinal cord. **(A,B)** Overview of 5-ethynyl-2′-deoxyuridine (EDU) signal in spinal cords in non-runners **(A)** and runners **(B)**, and EDU-positive cells are indicated by arrows. **(C–G)** EDU-positive cells in runners are mostly Olig2-positive and about 4.8-fold more abundant in runners than in non-runners. Each circle represents the data from one animal. **P < 0.01; *n* = 4 in each group.

BrdU studies showed the proliferation and differentiation of glial progenitors in the intact adult rat spinal cord (Horner et al., 2000). To assess whether running affects cell proliferation in spinal cords, we injected EDU to label newly-born cells in 12-week runner and non-runner mutant animals. In non-runners, a few EDU-labeled cells were scattered in the gray and white matter of C5–C7 spinal segments (Figure 6A). In contrast, the number of EDU-labeled cells was significantly increased after 12-week exercise (Figure 6B), about 4.8-folds higher than in non-runners (Figure 6G). To trace the lineage of these EDU-positive cells, we coupled EDU staining with immunostaining for neuronal, oligodendrocyte, astrocyte and microglial cell markers and found that more than 90% of EDU labeled cells were positive for Olig2, an oligodendrocyte marker (Figures 6C–F; *p* = 0.001; *t* = 5.63; *df* = 6).

### Running Exercise Modifies Transcriptomic Profiles in the Spinal Cord

**Figure 7:**
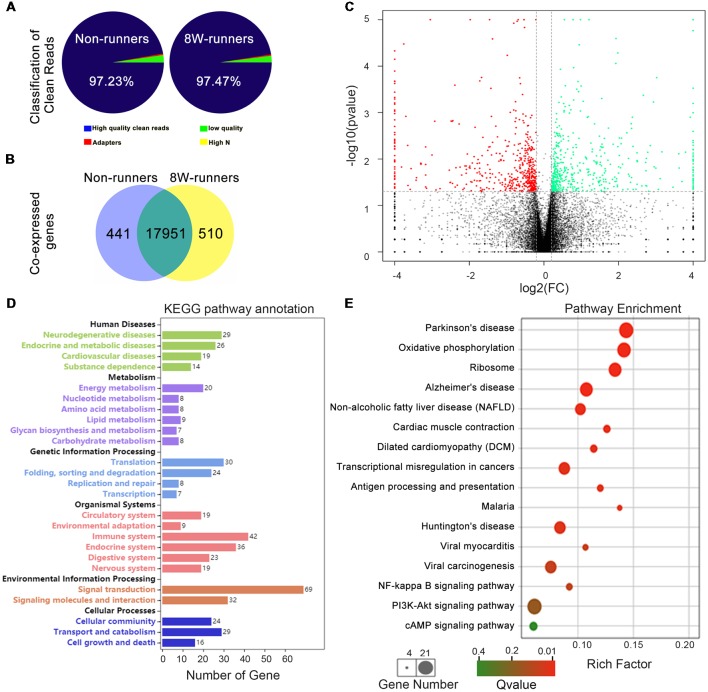
Transcriptomic profiling of mutant mice after training. **(A)** Classification of clean reads in non-runner and 8-week (8W) runner mutants. **(B)** There are a total number of 17,951 co-expressed genes in runner and non-runner mutants, 441 unique genes in non-runners, and 510 unique genes in runners. **(C)** Scatter plots show co-expressed genes in runner and non-runner mutants. Green dots indicate down-regulated genes, and red dots represent up-regulated genes in the runner group, whereas brown dots show genes with no significant changes. Cutoffs of a fold change (FC) at 1.15, and *P*-value at less than 0.05 are considered significant. **(D)** Differentially expressed genes (DEGs) annotated in KEEG pathways. **(E)** KEGG pathway enrichment of the DEGs. The rich factor is calculated using the number of enriched genes divided by the number of all background genes in the corresponding pathway.

To further explore potential molecular mechanisms involved in exercise-induced neural plasticity in mutant animals, we compared gene expression of spinal C5–C7 segments in 8-week runners and matched non-runners, using RNAseq. mRNA libraries were generated from runners and non-runners mutant mice (three repeats for each group). High quality clean reads of 30, 851, 978 (97.23% of raw reads) were obtained for non-runners and 29, 818, 423 (97.47% of raw reads) for runners (Figure 7A). In the runner and non-runner groups, 88.30% and 88.60% of total clean reads were uniquely matched to gene-mapped reads, respectively. There were a total number of 17,951 co-expressed genes in runner and non-runner mutants, 441 unique genes in non-runners and 510 unique genes in runners (Figure 7B). Among them, 404 genes were up-regulated and 398 genes were down-regulated in the runner compared to the non-runner mutants (Figure 7C; *p* < 0.05). We then examined these 802 genes using pathway enrichment analysis and found that a total of 290 DEGs were annotated in the data bank (Supplementary Table S1). Some differentially activated pathways were related to neurodegenerative diseases (i.e., Parkinson’s, Alzheimer’s and Huntington’s diseases) and energy metabolism (especially oxidative phosphorylation; Figures 7D,E). Sixty-nine DEGs were involved in signal transduction, and three top signaling pathways in the KEGG map were the NF-κB signaling pathway (10 DEGs, such as *Xiap*, *Tnfrsf1a*, *Relb* and *Tab3*), the PI3K-Akt signaling pathway (21 DEGs, such as *Fgf1*, *Igf1*, *Epor*, and *Thbs1*) and the cAMP signaling pathway (12 DEGs, such as *Gipr, PLD1*, *Rock1*, *Epac1* and *Gria1*; Figures 7D,E; Supplementary Table S1).

### Exercise Induces Changes of Synaptic and Myelin-Related Proteins, but Not of Neurotrophins or Cytokines in Spinal Cords

**Figure 8:**
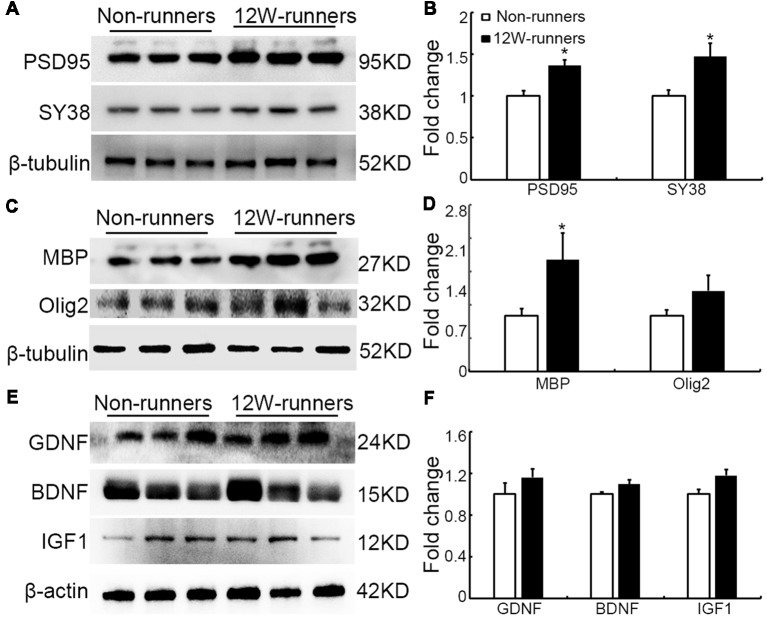
Expression of synaptic and myelin-related proteins is increased after training. **(A,B)** Western blot analysis and quantification of postsynaptic density protein (PSD95) and the presynaptic protein synaptophysin (SY38). **(C,D)** Analysis of oligodendrocyte transcription factor-2 (Olig2) and myelin basic protein (MBP). **(E,F)** Neurotrophins glia-derived neurotrophic factor (GDNF), brain-derived neurotrophic factor (BDNF) and insulin like growth factor-1 (IGF-1). β-tubulin or β-actin is used as control as indicated. *P < 0.05; three animals used in each group.

Results mentioned above suggest that increased descending axons (rubrospinal and monoaminergic axons) may make more synapses with spinal neurons after training. To document this further, the levels of PSD95 and presynaptic protein synaptophysin (SY38) were analyzed by western blot in extracts from C5 to C7 spinal samples. After 12 weeks of exercise, PSD95 and SY38 proteins were significantly increased in runners compared to non-runners, respectively 1.37 and 1.60-fold (Figures 8A,B; *p* = 0.015, *t* = 4.059 and *p* = 0.049, *t* = 2.79, *df* = 4, respectively). Although this is somewhat controversial (Duncan et al., 2018), re-myelination of axonal sheaths may assist functional recovery after injury (Takase et al., 2018; Yang et al., 2018). In line with increased oligodendrogenesis after running exercise, we found that the protein level of MBP was significantly increased in 12-weeks runners, 2.01-folds of that in non-runners (Figures 8C,D; *p* = 0.03, *t* = 6.71, *df* = 4). In addition, Olig2 expression also showed an increased trend in runner animals (Figures 8C,D).

**Figure 9:**
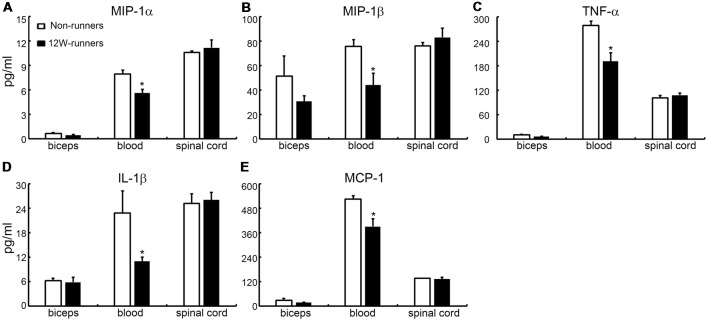
Cytokine levels in spinal cord are unchanged by training. **(A–E)** Exercise can decrease the content of five inflammatory factors in the blood (MIP-1β; TNF-α; IL-1β; MCP-1; MIP-1α), but not in the muscle or spinal cord. *P < 0.05; three animals used in each group.

It is widely believed that neurotrophic factors play a significant role in neural plasticity and neurogenesis after exercise (Cobianchi et al., 2017). We therefore assessed the expression of a few neurotrophic factors including BDNF, GDNF and IGF1 in C5–C7 spinal samples from 12-week runners and non-runners. Quite unexpectedly, we did not find any significant difference in BDNF, GDNF and IGF1 protein levels in both groups (Figures 8E,F; *P* > 0.05; *n* = 3 animals in each group). To further identify whether running exercise induced changes of inflammatory cytokine levels, we compared samples from blood, muscles and C5–C7 spinal tissues in 12-week runner mice and matched non-runner mice, using Bioplex. There was a significant decrease of MIP-1β, TNF-α, IL-1β, MCP-1 and MIP-1α in blood of the running group, but not in muscles or spinal cord extracts (Figure 9; *n* = 3 animals in each group).

## Discussion

The present study was designed to determine whether voluntary exercise is beneficial and improves locomotor plasticity in a mutant mouse model with congenital absence of the CST. Our results show that training for 8–12 consecutive weeks improves fine motor skills and muscle force. This is correlated with: (i) increased ramifications of rubrospinal and monoaminergic axons in the spinal cord, and increased contacts between dopaminergic varicosities and motor neurons; (ii) stimulation of spinal oligodendrogenesis and increased expression of synaptic and myelin marker proteins; (iii) modified expression of various genes and signaling pathways detected using transcriptomics analysis; and (iv) absence of detectable modifications of growth factors and cytokine expression in spinal cord.

The CST and rubrospinal tract are two most important descending axonal bundles that control spinal motor neurons to drive limb movements, and may partly palliate each other (Kennedy, 1990; Cheney et al., 1991). The observation that *Emx1-Cre;Celsr3^f/-^* mice have no CST and poor fine movement control shows that skilled motor function (such as food pellet taking) is especially dependent on the CST and cannot be spontaneously compensated by rubrospinal input (Han et al., 2015). Here we provide evidence that voluntary wheel training can rescue fine movement control in CST-deficient mutant mice, thus providing evidence for intrinsic plasticity in the absence of lesion. Our results show a significant increase of rubrospinal terminals in spinal segments in runner compared to non-runner mutants, and exercise resulted in increased spinal cord expression of synaptic proteins PSD-95 and SY38. Exercise may thus trigger formation of additional synapses by rubrospinal axons, which could serve a function comparable to that of the CST in fine movement control. This should be confirmed using transynaptic labeling, for example using modified rabies virus (Callaway and Luo, 2015).

Fine movements of forelimbs are also dependent on coordinated monoaminergic inputs to spinal neurons. Serotoninergic inputs from parapyramidal raphe nuclei facilitate the initiation of movements (Jordan et al., 2008; Cabaj et al., 2017) and activate central pattern generators that produce rhythmic outputs, even after the loss of the CST (Schmidt and Jordan, 2000). Similarly, dopaminergic inputs from the A11 diencephalic region modulate locomotor function (Sharples et al., 2015), and motor skill learning is impaired upon inhibition of dopaminergic signaling (Willuhn and Steiner, 2008). In our study, the input of monoaminergic axons to spinal cord was increased and this may contribute to coordinating the activity of spinal motor neurons and/or segmental interneurons.

Spinal motor axons are the final pathway from upper motor neurons to muscles and drive muscle activity via neuromuscular junctions. After exercise, muscle strength and weight were augmented; neuromuscular junctions were increased in number, and muscle innervation became more efficient, as indicated in electromyographic recordings. These changes presumably reflect increased supraspinal inputs to spinal motor neurons (Ding et al., 2014), and/or a more efficient feedback loop between muscles and spinal motorneurons after wheel running training.

After wheel running, we found a significant increase of oligodendrogenesis with new myelin formation indicated by elevated MBP protein levels. Previous studies showed that, in rodents, active central myelination is required for skill motor learning (McKenzie et al., 2014). In mice submitted to wheel running, a secondary wave of oligodendrocyte precursor proliferation and differentiation is present in motor cortex (Xiao et al., 2016), and structural changes of the white matter were observed by MRI after learning a novel motor skill (Sampaio-Baptista et al., 2013). The role of re-myelination after SCI is somewhat controversial: some studies suggest that it is important for functional improvement (Karimi-Abdolrezaee et al., 2006; Biernaskie et al., 2007), whereas another work proposed that locomotor recovery does not require oligodendrocyte remyelination (Duncan et al., 2018). In intact adult spinal cords, cell proliferation is rare and restricted to the surrounding of the central canal (Barnabé-Heider et al., 2010; Becker et al., 2018). In runner mutants, we found EDU-labeled cells widely scattered in the gray and white matter, indicating that they undergo migration and may foster new myelin formation of long projecting and local spinal axons. Altogether, we propose that, in our mutant mice, running-induced myelination modulates neural spinal networks to foster skilled motor performance.

Several molecules and signaling mechanisms are thought to be involved in exercise-induced neural plasticity, such as neurotrophic factors (Zhao et al., 2012) or cAMP signaling (Ko et al., 2018). Here, we provide evidence that wheel-running influences the transcriptomic profile in the spinal cord. Wheel running is a moderate intervention and its effect may be highly dynamic. After 8-week running, expression of several genes was modified, and regulation of some signaling pathways were predicted, including pathways related to neuron degeneration (i.e., Parkinson’s disease), oxidative phosphorylation, NF-κB signaling, PI3K-Akt signaling, and cAMP signaling. As described before, wheel running induces a significant elevation of dopaminergic fiber ramification and TH protein levels in spinal cord, and this may be a reason why some DEGs are associated with Parkinson’s disease. Those data also suggest that the dopaminergic system might be implicated in exercise-induced neural plasticity. Oxidative phosphorylation is critical for ATP production by mitochondria, and increased mitochondrial metabolism contributes to neural plasticity and cell proliferation (Radak et al., 2007; Batty et al., 2017). The observed changes of oxidative phosphorylation-related genes presumably favor exercise-induced axonal ramification, new synapse formation and oligodendrogenesis.

We found 12 DEGs involved in the cAMP signaling pathway, among which *Gipr*, *PLD1*, *Rock1*, *Epac1* and *Gria1* are involved in neural plasticity and/or neurite growth (Mead and Stephens, 2003; Zhang et al., 2004; Faivre et al., 2011; Yang et al., 2012; Greathouse et al., 2018). In addition, 20 DEGs are involved in the PI3K/Akt pathway, and 10 DEGs in NF-κB signaling. These genes may point to potential mechanisms of exercise-induced locomotor plasticity that remain to be studied further.

Physical exercise results in increased production of neurotrophic factors and regulation of levels of some cytokines, cytokine inhibitors and chemokines, all of which may contribute to improving motor function and cognition in neurodegenerative disorders (Svensson et al., 2015; Wei et al., 2018). In SCI, local levels of cytokines critically influence the kinetics of neuron survival, axon degeneration and regeneration (Sun et al., 2016). Unexpectedly, we found a downregulation of pro-inflammatory cytokines including TNF-α, IL-1β, MCP-1, MIP-1α and MIP-1β after 12-week running only in serum but not in the spinal cord or muscles. Furthermore, we could not detect any differences in levels of classic neurotrophic factors including BDNF, GDNF and IGF1 after 12-week running exercise. The reasons for this apparent discrepancy are not clear. Spinal cord cytokine and growth factor levels may be more prone to change in the context of a lesion than upon exercise alone. Also, we measured cytokines and growth factors only at the end of the exercise period (12 weeks), whereas changes could follow a dynamic course.

In *Emx1-Cre;Celsr3^f/-^* mutant mice, the CST never develops, a situation which is different from experimental lesions. In that sense, those mice do not provide a fully relevant model for SCI or cerebral palsy (Clowry et al., 2014). With that reservation, our observations that motor-skill learning exercise can trigger intrinsic motor plasticity, even in the absence of lesion, is strong indication that similar procedures should be helpful in lesion cases. Our results suggest that skilled motor learning training, used as a complement to treadmill training, should be beneficial to patients with SCI, cerebral palsy and related conditions.

## Data Availability

The datasets generated for this study can be found in SRA accession, PRJNA521582.

## Author Contributions

LZ, YQ and K-FS designed the experiment. WZ, BY, HW, TL and PY performed the research. WZ and LS analyzed the data. WZ and LZ wrote the manuscript.

## Conflict of Interest Statement

The authors declare that the research was conducted in the absence of any commercial or financial relationships that could be construed as a potential conflict of interest.

## Footnotes

^1^http://www.genomics.cn
